# The clinical impact and dissemination of carbapenemase-producing *Enterobacter*: a genome-based study in China

**DOI:** 10.1128/spectrum.01166-25

**Published:** 2025-08-12

**Authors:** Shikai Wu, Yu Feng, Yanling He, Yuling Xiao, Yi Xie, Hongxia Wen, Li Wei, Wenjing Wu, Chengcheng Wang, Alan McNally, Zhiyong Zong

**Affiliations:** 1 Center of Infectious Diseases, West China Hospital, Sichuan University 12530, Chengdu, China; 2 Division of Infectious Diseases, State Key Laboratory of Biotherapy, Chengdu, China; 3 Laboratory of Pathogen Research, West China Hospital, Sichuan University 12530, Chengdu, China; 4 Department of Laboratory Medicine, West China Hospital, Sichuan University 12530, Chengdu, China; 5 Department of Laboratory Medicine, West China Second Hospital, Sichuan University 12530, Chengdu, China; 6 Department of Infection Control, West China Hospital, Sichuan University 12530, Chengdu, China; 7 School of Infection Inflammation & Immunology, College of Medicine and Health, University of Birmingham 1724, Birmingham, United Kingdom; 8 State Key Laboratory of Respiratory Health and Multimorbidity, Chengdu, China; Jinan University, Shenzhen, China

**Keywords:** *Enterobacter*, population structure, carbapenem resistance, carbapenemase, dissemination

## Abstract

Carbapenemase-producing *Enterobacter* (CPEn) has globally emerged to be a severe threat to human health. However, the disease spectrum, impact on affected patients’ outcomes, and transmission of CPEn are largely uncharacterized. We performed genome sequencing on a collection of CPEn clinical isolates spanning a six-year period, collating corresponding disease spectrum data and patient prognosis. Among the 128 sequenced CPEn, 2/3 were clinical isolates from a range of infections with bloodstream infections the most common. The remaining isolates were obtained from patient screening and considered to be colonizing the patients. Most (77.6%) CPEn infection isolates were healthcare associated. The predicted in-hospital mortality rate of patients with CPEn infections was 32.9%. We extended our analysis to all publicly available *Enterobacter* genomes in China to uncover the population structure and transmission of CPEn by phylogenomic analysis. There were 562 CPEn isolated in China among the 14,648 publicly available *Enterobacter* genomes. Consistent with our local isolates, ST171 *E. xiangfangensis* encoding *bla*_NDM-1_ or *bla*_NDM-5_ was the most common CPEn type in hospitals across China. Intra-hospital transmission and several inter-hospital and cross-region transmission/strain-movement events of ST171 CPEn were observed. In conclusion, CPEn typically causes healthcare-associated infection and is a severe clinical problem. Countermeasures may need to focus on patient transfer and environmental cleaning including sinks.

**IMPORTANCE:**

Carbapenemase-producing *Enterobacter* (CPEn) causes difficult-to-treat infections and has emerged globally as a significant antimicrobial resistance threat. Here, we generated genome sequences of 128 CPEn clinical isolates with accompanying clinical data. We found that CPEn causes a variety of infections, typically healthcare associated, and also asymptomatically colonizes patients. Among infections due to CPEn, bacteremia, pneumonia, and urinary tract infection are the most common. CPEn infections lead to a high predicted in-hospital all-cause mortality rate (32.9%). We examined all publicly-available *Enterobacter* genomes, identified an additional 562 CPEn strains from China, and unveiled the complex population structure of CPEn. We identified multiple intra- and inter-ward transmissions in the hospital and uncovered several inter-hospital and cross-region dissemination of CPEn. Infection control is key to counter CPEn and may need to include enhanced environmental hygiene and measures to reduce transmission related to patient transfer.

## INTRODUCTION

*Enterobacter* is a genus of the *Enterobacteriaceae* family and is widely distributed in nature (1). *Enterobacter* is part of the commensal microflora of the human gastrointestinal tract and comprises a number of species able to cause human infections, which are commonly termed as the *Enterobacter cloacae* complex within clinical settings (2). Carbapenems are one of the major options for treating severe infection caused by *Enterobacter*. However, resistance to carbapenems has arisen rapidly over the last decade, and *Enterobacter* has become a common carbapenem-resistant microorganism second only to *Escherichia coli* and *Klebsiella pneumoniae* (3). Carbapenem resistance in *Enterobacteriaceae* is mainly due to production of carbapenem-hydrolyzing β-lactamases (carbapenemases). Carbapenemase-producing *Enterobacter* (CPEn) was first described in the 1990s (4) and is now globally reported. A number of carbapenemases including Ambler Class A (e.g., KPC and IMI), B (NDM, IMP, and VIM), and D (e.g., OXA-48 and OXA-181) have been found in CPEn. Compared to carbapenemase-producing *E. coli* and *K. pneumoniae*, CPEn is understudied, hindering effective management and prevention of CPEn infections. In particular, the disease spectrum, impact on affected patients’ outcomes, and detailed in-hospital transmission dynamics of CPEn, as well as the population structure and the dissemination of CPEn across countries, remain largely unclear.

To address the gap, we performed a hybrid study. First, we performed a longitudinal genome study of a six-year collection of *Enterobacter* clinical isolates aligning with patients’ records to examine the disease spectrum of CPEn, the corresponding impact on prognosis, and in-hospital clonal transmission. Second, we extended our analysis to all publicly available *Enterobacter* genomes and uncovered the population structure and national transmission of CPEn in China. We also performed dating analysis for the most common lineage of CPEn, attempting to understand its emergence. Furthermore, we linked our CPEn isolates with other Chinese isolates by assigning transmission clusters. The generated results provide much-needed insights which will inform the design and implementation of effective infection control practices for this critical pathogen.

## MATERIALS AND METHODS

### Setting and isolates

*Enterobacter* isolates were recovered from clinical samples as part of routine care for patients with infections between June 2016 and July 2022. For patients with more than one isolate, we included the earliest one. Preliminary species identification and carbapenem susceptibility testing were performed using the VITEK II microbiological system (bioMérieux, Marcy-l'Étoile, France) with interpretation of susceptibility categories according to the Clinical and Laboratory Standards Institute (CLSI) 2023 (5). This study was approved by the ethical committee of West China Hospital with informed consent being waived. Patients were consented with the care provided by the hospital and its staff, which included sample collection for managing their infection or suspected infection. All patient data were anonymized.

### Clinical data and definition

*Enterobacter* isolates were considered pathogens causing infection if any of the following criteria applied: (i) Isolate was recovered from typically sterile body fluids such as blood, cerebrospinal fluid, pleural fluid, and ascites; (ii) from patients with symptoms of urinary tract infections accompanied by the bacterial load of ≥10^5^ colony-forming units (CFU)/mL or <10^5^ CFU/mL but in the absence of any other pathogens detected; or (iii) from respiratory tract samples or secretions in patients with the corresponding infection diagnosis but without other pathogens detected. Blood stream infections were classified into primary or secondary types depending on whether *Enterobacter* isolates were obtained from additional sites. Infection caused by an isolate recovered from a sample collected 48 h after admission is considered healthcare associated. Prognosis (death, predicted to die when discharged at patients’ own will, or recovery) was determined by two investigators. The hospitalization data (date, ward, room, and bed number) of patients with an isolate belonging to a clone comprising ≥5 isolates were retrieved, reviewed, and plotted in a time-informed diagram.

### Whole-genome sequencing

Bacterial genomic DNA was extracted using the QIAamp DNA mini kit (Qiagen, Hilden, Germany) and was subjected to whole-genome sequencing with a 150 bp pair-end layout, aiming for at least 200 × sequencing coverage using the NovaSeq 6000 Sequencing System (Illumina, San Diego, CA). Sequencing adapters were first trimmed by Trimmomatic v0.39 (6), and reads were assembled into contigs using SPAdes v3.15.5 (7). Draft genome sequences of our isolates have been deposited in NCBI with accession no. listed in Table 1. Assemblies and sequencing raw data from Sequence Read Archive (SRA) of Taxonomy *Enterobacter* (accessed by 30 September 2023) were retrieved from National Center for Biotechnology Information (NCBI). SRA derived fastq files were processed as above.

**TABLE 1:** The 128 CPEn isolates recovered in West China Hospital^*a*^

Isolate	Assembly	Species	ST	Carbapenemase	Source	Date	Ref.
020122	GCA_003964705.1	*E. asburiae*	25	NDM-1	Sputum	Jan-17	
155105	GCA_025502525.1	*E. asburiae*	1587	FRI-11	Secretion	Nov-21	(8)
090029	GCA_003965205.1	*E. bugandensis*	718	NDM-5	Blood	Sep-17	(9)
155062	JAXHHS000000000	*E. chengduensis*	414	NDM-1	Sputum	Apr-21	
170220	JAXHGV000000000	*E. chengduensis*	414	NDM-1	Blood	Jun-22	
090005	GCA_003965415.1	*E. cloacae*	1	NDM-1	Blood	Jun-17	(9)
155007	JAXHIV000000000	*E. cloacae*	432	NDM-1	Sputum	Feb-20	
120098	JAXHJX000000000	*E. hoffmannii*	78	NDM-1	Respiratory	Nov-19	
170268	JAXHGO000000000	*E. hoffmannii*	78	NDM-1	Respiratory	Jul-22	
120132	JAXHJU000000000	*E. hoffmannii*	97	IMP-4; NDM-1	Sputum	Jan-20	
020029	GCA_003965735.1	*E. hoffmannii*	97	NDM-1	Sputum	Jul-16	
140043	JAXHJF000000000	*E. hoffmannii*	97	NDM-1	Sputum	Jul-19	
140048	JAXHJC000000000	*E. hoffmannii*	97	NDM-1	Respiratory	Aug-19	
120125	JAXHJW000000000	*E. hoffmannii*	97	NDM-1	Respiratory	Feb-20	
155070	JAXHHL000000000	*E. hoffmannii*	97	NDM-1	Sputum	Sep-21	
155053	JAXHHY000000000	*E. hoffmannii*	118	NDM-1	Sputum	Mar-21	
155068	JAXHHN000000000	*E. hoffmannii*	152	NDM-1	Sputum	Jun-21	
170199	JAXHGY000000000	*E. hoffmannii*	419	NDM-1	Blood	Jun-22	
155066	JAXHHP000000000	*E. hoffmannii*	968	NDM-1	Catheter tip	May-21	
120129	JAXHJV000000000	*E. hoffmannii*	1373	NDM-1	Respiratory	Feb-20	
090099	JAXHKF000000000	*E. kobei*	591	NDM-1	Urine	Oct-18	
120040	JAXHKA000000000	*E. kobei*	591	NDM-1	Blood	Feb-19	
140044	GCA_031460405.1	*E. soli*	–	IMP-4	Blood	Aug-19	
120085	JAXHJZ000000000	*E. xiangfangensis*	51	NDM-5	Blood	May-19	
155004	JAXHIY000000000	*E. xiangfangensis*	51	NDM-5	Pleural fluid	Feb-20	
155001	CP139567	*E. xiangfangensis*	51	NDM-5	Sputum	Feb-20	
155006	JAXHIW000000000	*E. xiangfangensis*	51	NDM-5	Sputum	Feb-20	
155010	JAXHIU000000000	*E. xiangfangensis*	51	NDM-5	Blood	Apr-20	
155050	JAXHIA000000000	*E. xiangfangensis*	51	NDM-5	Sputum	Feb-21	
155058	JAXHHV000000000	*E. xiangfangensis*	51	NDM-5	Ascites	Mar-21	
155067	JAXHHO000000000	*E. xiangfangensis*	51	NDM-5	Respiratory	May-21	
170183	JAXHHA000000000	*E. xiangfangensis*	51	NDM-5	Sputum	Jun-22	
155075	JAXHHH000000000	*E. xiangfangensis*	88	NDM-1	Urine	Oct-17	
140031	JAXHJL000000000	*E. xiangfangensis*	88	NDM-1	Urine	Apr-19	
140037	JAXHJJ000000000	*E. xiangfangensis*	88	NDM-1	Secretion	Jun-19	
140041	JAXHJH000000000	*E. xiangfangensis*	88	NDM-1	Secretion	Jun-19	
155005	JAXHIX000000000	*E. xiangfangensis*	88	NDM-1	Blood	Feb-20	
155037	JAXHIK000000000	*E. xiangfangensis*	90	IMP-4	Urine	Nov-20	
155054	JAXHHX000000000	*E. xiangfangensis*	90	NDM-1	Urine	Mar-21	
040006	GCA_002850625.1	*E. xiangfangensis*	93	IMP-1; NDM-1	Urine	Mar-17	
090085	JAXHKK000000000	*E. xiangfangensis*	114	NDM-1	Secretion	Feb-18	
090096	JAXHKH000000000	*E. xiangfangensis*	114	NDM-1	Sputum	Sep-18	
155030	JAXHIL000000000	*E. xiangfangensis*	114	NDM-1	Sputum	Oct-20	
170239	JAXHGR000000000	*E. xiangfangensis*	114	NDM-1	Blood	Jul-22	
040008	GCA_003965455.1	*E. xiangfangensis*	114	NDM-5	Secretion	Mar-17	
170215	JAXHGW000000000	*E. xiangfangensis*	114	NDM-5	Sputum	Jun-22	
090100	JAXHKE000000000	*E. xiangfangensis*	116	NDM-1	Sputum	Oct-18	
140028	JAXHJN000000000	*E. xiangfangensis*	116	NDM-1	Sputum	Feb-19	
120089	GCA_023887695.1	*E. xiangfangensis*	116	NDM-1	Blood	Jun-19	
140042	JAXHJG000000000	*E. xiangfangensis*	116	NDM-1	Blood	Jun-19	
140035	JAXHJK000000000	*E. xiangfangensis*	116	NDM-1	Sputum	Jun-19	
120156	JAXHJT000000000	*E. xiangfangensis*	116	NDM-1	Blood	Aug-19	
140057	JAXHJB000000000	*E. xiangfangensis*	116	NDM-1	Sputum	Nov-19	
155046	JAXHIC000000000	*E. xiangfangensis*	116	NDM-1	Sputum	Jan-21	
155061	JAXHHT000000000	*E. xiangfangensis*	116	NDM-1	Sputum	Apr-21	
155069	JAXHHM000000000	*E. xiangfangensis*	120	NDM-5	Secretion	Sep-21	
090062	GCA_003964445.1	*E. xiangfangensis*	133	NDM-5	Sputum	Jun-18	
155081	JAXHHF000000000	*E. xiangfangensis*	134	NDM-1	Sputum	Jun-19	
155018	JAXHIQ000000000	*E. xiangfangensis*	134	NDM-1	Catheter tip	Jun-20	
140534	JAXHJA000000000	*E. xiangfangensis*	134	NDM-1	Rectal swab	Jun-20	
155042	JAXHIG000000000	*E. xiangfangensis*	134	NDM-1	Ascites	Jan-21	
155072	JAXHHJ000000000	*E. xiangfangensis*	134	NDM-1	Blood	Oct-21	
090103	JAYGOH000000000	*E. xiangfangensis*	171	KPC-2; NDM-5	Respiratory	Jun-18	
090068	GCA_003964355.1	*E. xiangfangensis*	171	NDM-1	Urine	Jun-16	
045001	GCA_003964795.2	*E. xiangfangensis*	171	NDM-1	Blood	Jan-18	(9)
155080	JAXHHG000000000	*E. xiangfangensis*	171	NDM-1	Sputum	Feb-19	
140030	JAXHJM000000000	*E. xiangfangensis*	171	NDM-1	Urine	Mar-19	
140046	JAXHJE000000000	*E. xiangfangensis*	171	NDM-1	Ascites	Aug-19	
140047	JAXHJD000000000	*E. xiangfangensis*	171	NDM-1	Urine	Aug-19	
155026	JAXHIN000000000	*E. xiangfangensis*	171	NDM-1	Urine	Sep-20	
155028	JAXHIM000000000	*E. xiangfangensis*	171	NDM-1	Secretion	Oct-20	
155039	JAXHIJ000000000	*E. xiangfangensis*	171	NDM-1	Urine	Dec-20	
155045	JAXHID000000000	*E. xiangfangensis*	171	NDM-1	Pleural fluid	Jan-21	
155044	JAXHIE000000000	*E. xiangfangensis*	171	NDM-1	Urine	Jan-21	
155051	JAXHHZ000000000	*E. xiangfangensis*	171	NDM-1	Secretion	Feb-21	
170177	JAXHHB000000000	*E. xiangfangensis*	171	NDM-1	Blood	Apr-22	
170194	JAXHGZ000000000	*E. xiangfangensis*	171	NDM-1	Blood	Jun-22	
155074	JAXHHI000000000	*E. xiangfangensis*	171	NDM-5	Respiratory	Aug-17	
090011	GCA_003965345.2	*E. xiangfangensis*	171	NDM-5	Blood	Oct-17	(9)
016162	GCA_003964435.1	*E. xiangfangensis*	171	NDM-5	Rectal swab	Jan-18	
090084	JAXHKL000000000	*E. xiangfangensis*	171	NDM-5	Sputum	Jan-18	
090022	GCA_003965265.1	*E. xiangfangensis*	171	NDM-5	Blood	Feb-18	(9)
090023	GCA_003965255.1	*E. xiangfangensis*	171	NDM-5	Blood	Feb-18	(9)
090055	GCA_003964535.1	*E. xiangfangensis*	171	NDM-5	Blood	Mar-18	(9)
090101	JAXHKD000000000	*E. xiangfangensis*	171	NDM-5	Sputum	Mar-18	
090087	JAXHKJ000000000	*E. xiangfangensis*	171	NDM-5	Respiratory	Apr-18	
090059	GCA_003964815.1	*E. xiangfangensis*	171	NDM-5	Blood	May-18	(9)
090091	JAXHKI000000000	*E. xiangfangensis*	171	NDM-5	Urine	Jun-18	
090076	GCA_003964265.1	*E. xiangfangensis*	171	NDM-5	Blood	Oct-18	
120031	JAXHKB000000000	*E. xiangfangensis*	171	NDM-5	Blood	Jan-19	
140023	JAXHJO000000000	*E. xiangfangensis*	171	NDM-5	Blood	Jan-19	
120090	JAXHJY000000000	*E. xiangfangensis*	171	NDM-5	Blood	Jun-19	
120163	JAXHJS000000000	*E. xiangfangensis*	171	NDM-5	Blood	Aug-19	
120179	JAXHJQ000000000	*E. xiangfangensis*	171	NDM-5	Blood	Nov-19	
120185	JAXHJP000000000	*E. xiangfangensis*	171	NDM-5	Blood	Nov-19	
155003	JAXHIZ000000000	*E. xiangfangensis*	171	NDM-5	Ascites	Feb-20	
155012	JAXHIT000000000	*E. xiangfangensis*	171	NDM-5	Secretion	Apr-20	
155017	JAXHIR000000000	*E. xiangfangensis*	171	NDM-5	Secretion	Jun-20	
155020	JAXHIP000000000	*E. xiangfangensis*	171	NDM-5	Sputum	Jul-20	
155040	JAXHII000000000	*E. xiangfangensis*	171	NDM-5	Sputum	Jan-21	
155063	JAXHHR000000000	*E. xiangfangensis*	171	NDM-5	Ascites	May-21	
155064	JAXHHQ000000000	*E. xiangfangensis*	171	NDM-5	Catheter tip	May-21	
155071	JAXHHK000000000	*E. xiangfangensis*	171	NDM-5	Respiratory	Oct-21	
170232	JAXHGT000000000	*E. xiangfangensis*	171	NDM-5	Sputum	Jun-22	
170235	JAXHGS000000000	*E. xiangfangensis*	171	NDM-5	Sputum	Jun-22	
170231	JAXHGU000000000	*E. xiangfangensis*	171	NDM-5	Urine	Jun-22	
170257	JAXHGP000000000	*E. xiangfangensis*	171	NDM-5	Urine	Jul-22	
090070	GCA_003964755.1	*E. xiangfangensis*	177	NDM-1	Urine	Oct-16	
155015	JAXHIS000000000	*E. xiangfangensis*	177	NDM-1	Secretion	May-20	
155043	JAXHIF000000000	*E. xiangfangensis*	177	NDM-1	Urine	Jan-21	
170255	JAXHGQ000000000	*E. xiangfangensis*	177	NDM-1	Urine	Jul-22	
155082	JAXHHE000000000	*E. xiangfangensis*	182	NDM-1	Sputum	Aug-19	
155083	JAXHHD000000000	*E. xiangfangensis*	182	NDM-1	Sputum	Nov-20	
155088	JAXHHC000000000	*E. xiangfangensis*	182	NDM-1	Urine	Mar-22	
155041	JAXHIH000000000	*E. xiangfangensis*	190	NDM-5	Sputum	Jan-21	
155055	JAXHHW000000000	*E. xiangfangensis*	190	NDM-5	Secretion	Mar-21	
155060	JAXHHU000000000	*E. xiangfangensis*	190	NDM-5	Sputum	Apr-21	
090069	GCA_003964275.1	*E. xiangfangensis*	295	NDM-1	Urine	Aug-16	
170203	JAXHGX000000000	*E. xiangfangensis*	350	NDM-1	Secretion	Jun-22	
020038	GCA_003428425.1	*E. xiangfangensis*	418	NDM-5	Sputum	Oct-16	
090013	GCA_003965675.1	*E. xiangfangensis*	418	NDM-5	Blood	Nov-17	(9)
120030	JAXHKC000000000	*E. xiangfangensis*	418	NDM-5	Blood	Dec-18	
090097	JAXHKG000000000	*E. xiangfangensis*	421	NDM-1	Sputum	Sep-18	
090075	GCA_003964845.1	*E. xiangfangensis*	527	NDM-1	Sputum	Jun-18	
155021	JAXHIO000000000	*E. xiangfangensis*	566	NDM-1	Sputum	Jul-20	
090057	GCA_003964915.1	*E. xiangfangensis*	2651	NDM-1	Blood	Apr-18	(9)
140039	JAXHJI000000000	*E. xiangfangensis*	–	NDM-1	Sputum	Mar-19	
155048	JAXHIB000000000	*E. xiangfangensis*	–	NDM-5	Sputum	Feb-21	

^*a*^ “–” means undetectable in the current ST classification. If there are no references, the corresponding table cells are left empty.

### Genome-based analysis for taxonomy, sequence types, and antimicrobial resistance genes

Average nucleotide identity (ANI) between genomes and the type strains of *Enterobacter* species (Table S1) were calculated using FastANI v1.33 (10), with the threshold of 96% as the delineator to confirm species identity. The sequence type (ST) was determined using mlst v2.23.0 (https://github.com/tseemann/mlst) with the *E. cloacae* scheme curated in PubMLST (11). Antimicrobial resistance determinants were identified using AMRFinderPlus v3.10 (12) with a threshold of 60% coverage and 98% identity (13). Notably, we focused on acquired carbapenemases and excluded IMI/NCM-A type carbapenemases from analysis, which are typically chromosome-borne and remain rare (14–16).

### SNP calling and phylogenomic analysis

Genomes with the highest N*50* for each identified ST were selected as the reference genome for each ST and quality-controlled reads were mapped onto the appropriate reference using Snippy v4.6.0 (https://github.com/tseemann/snippy) with default settings. A pseudo alignment was generated using the snippy-core script from which a phylogenomic tree was inferred using IQ-TREE v2.2.2.6 (17) with GTR + GAMMA model and 1,000 bootstraps, ignoring recombination sites identified by Gubbins v3.3.2 (18). Phylogenomic trees were visualized using iTOL v5 online (19).

### Identification of transmission clusters

The average nucleotide substitution rate of ST171 *Enterobacter* was calculated utilizing BactDating v1.1.2 (20), with the “mixedcarc” model and 10^7^ iterations of the Markov Chain Monte Carlo (MCMC). To investigate transmission among ST171 CPEn isolates, a most parsimonious transmission diagram was constructed based on the SeqTrack algorithm (21) using GraphSNP (22). A cutoff of 8 SNPs (twofold of the upper 95% HPD of the calculated annual substitution rate) multiplied by the number of years between any two isolates was used to assign the most parsimonious transmission clusters.

### Statistical analysis

Statistical analysis was performed using SPSS version 26.0.0.0 (IBM Analytics, Armonk, NY). *P* value < 0.05 was considered statistically significant. The *χ*² test or Fisher’s exact test was used to compare categorical variables, and continuous variables were compared using the independent samples *t*-test or Mann–Whitney *U* test, as appropriate.

## RESULTS

### The majority of our CPEn isolates belong to *E. xiangfangensis* or *E. hoffmannii* and encode NDM carbapenemases

**Fig 1:**
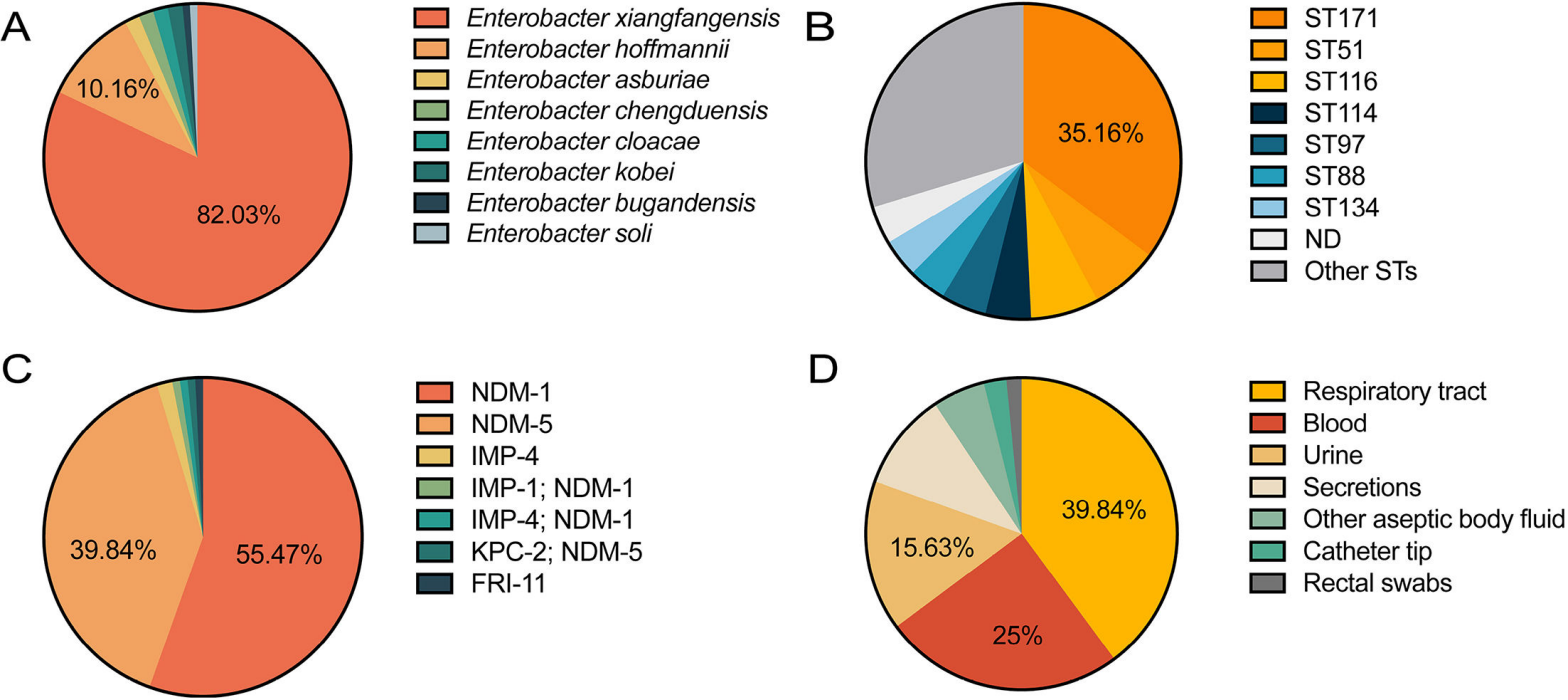
Characteristics of 128 CPEn recovered in the hospital between 2016 and 2022. (A) Precise species identification using FastANI. (B) Sequence type distribution of the 128 CPEn. (C) Carbapenemases identified by AMRFinder. (D) Sources of the 128 CPEn.

We collected and sequenced 373 *Enterobacter* isolates from clinical specimens of hospitalized patients including two rectal swabs collected due to abdominal distention or constipation. Among the 373 isolates, 128 were identified as CPEn. Most of these CPEn belonged to *E. xiangfangensis* (*n* = 105, 82.0%) or its closely related species (94.47% ANI between their type strains) *E. hoffmannii* (*n* = 13, 10.2%). The remaining 10 isolates were assigned to six species (one or two isolates for each) comprising *E. asburiae*, *E. bugandensis*, *E. chengduensis*, *E. cloacae*, *E. kobei*, and *E. soli* (Fig. 1). The carbapenemase positivity rate of *E. xiangfangensis* (105/200, 52.5%) is significantly higher than that of non-*E*. *xiangfangensis* (23/173, 13.29%; *P* ≤ 0.001). ST171 (*n* = 45, 35.2%) is the most common sequence type followed by ST51 (*n* = 9), ST116 (*n* = 9), ST114 (*n* = 6), and ST97 (*n* = 6) (Fig. 1). All but three CPEn (*n* = 125, 97.7%) carry *bla*_NDM_ encoding either NDM-1 (*n* = 73; two isolates also carry *bla*_IMP-1_ or *bla*_IMP-4_) or NDM-5 (*n* = 52; one isolate also carries *bla*_KPC-2_) (Fig. 1). The remaining three isolates carry either *bla*_IMP-4_ (*n* = 2; of *E. soli* or *E. xiangfangensis*) or *bla*_FRI-11_ (*n* = 1; of *E. asburiae*). Notably, all 45 ST171 (*E. xiangfangensis*) isolates have *bla*_NDM_, either *bla*_NDM-1_ (*n* = 14) or *bla*_NDM-5_ (*n* = 31).

### Clinical characteristics of CPEn infection and colonization

We examined the distribution, sample type, and infection site of CPEn in our hospital. The 128 isolates were recovered from patients in 25 wards, among which the Center Intensive Care Unit (CICU) was the most common (*n* = 32). CPEn isolates were mostly recovered from respiratory tract (*n* = 51, 39.8%), followed by blood (*n* = 32, 25.0%), urinary tract (*n* = 20, 15.6%), secretions (*n* = 13, 10.2%), ascites (*n* = 5), catheter tip (*n* = 3), pleural fluid (*n* = 2), and rectal swab (*n* = 2) (Fig. 1). We found that 85 (66.4%) CPEn isolates caused infections with bloodstream infections (*n* = 32) being the most common. We further examined bloodstream infections and uncovered that primary bloodstream infections were more common than secondary ones (25 vs 7 cases). The rest 53 infections were respiratory infection (*n* = 25), urinary infection (*n* = 12), skin and soft tissue infection (*n* = 9), intra-abdominal infection (*n* = 5), and pleural infection (*n* = 2). The remaining 43 (33.6%) CPEn colonization isolates were recovered from samples collected from the respiratory tract (*n* = 26), urinary tract (*n* = 8), skin (*n* = 3), catheter tip (*n* = 3), rectal swab (*n* = 2), and oral ulcer swab (*n* = 1). Patients with CPEn consisted of 97 men (75.8%) and 31 women (24.2%). Their median age was 54 years (Interquartile range [IQR], 40.75–70 years) with 43 patients (33.6%) older than 65 years. For the 85 cases with CPEn infection, the median time between admission and the day of the collection of the first CPEn-positive sample is 14 days (IQR, 3–27 days). Correspondingly, most (*n* = 66, 77.6%) CPEn infections were healthcare associated. There was no statistical difference between patients infected or colonized with CPEn by the gender (*P* = 0.915), age (*P* = 0.45), or ICU admission (*P* = 0.071). However, in respiratory specimens, the colonization group showed a higher proportion of CPEn compared to the infection group (*P* = 0.001). After CPEn infection, 57 patients recovered, 18 patients were predicted to die when discharged of their own will, and 10 patients died in hospital. The predicted in-hospital mortality rate of patients with CPEn infections, when combining patients who died with those predicted to die, was 32.9% with no statistically difference compared to those colonized with CPEn (34.9%, *P* = 0.686).

### CPEn are widely distributed across China with the vast majority of isolates encoding metallo-β-lactamases

We retrieved all available genome sequences, either assemblies (*n* = 8,459) or SRA files (*n* = 11,554), under the Taxonomy *Enterobacter* (accessed by 30 September 2023) from GenBank. After quality control (Fig. S1 for a flowchart exhibiting the inclusion and exclusion of the genomes), we finally included a total of 14,648 *Enterobacter* genomes comprising 4,145 assemblies and 10,503 SRA files. We identified 7,533 CPEn (51.4%) out of all 14,648 *Enterobacter* genomes based on the identification of carbapenemase genes. Among the 7,533 CPEn genomes, 562 (7.5%) originated from China (Data Set S1). The 562 CPEn consist of *E. xiangfangensis* (*n* = 418, 74.4%), *E. hoffmannii* (*n* = 70, 12.5%), *E. asburiae* (*n* = 27, 4.8%), *E. kobei* (*n* = 12, 2.1%), *E. hormaechei* (*n* = 10, 1.8%), *E. cloacae* (*n* = 8, 1.4%), *E. chengduensis* (*n* = 6, 1.1%), and four other *Enterobacter* species or taxa (*n* = 11) (Fig. S2). CPEn isolates were isolated in 27 of the 31 provincial regions on the mainland (Data Set S1 ), indicative of its wide distribution in China. About 1/4 of Chinese CPEn isolates sequenced were recovered in Zhejiang province (*n* = 140, 24.9%), followed by Jiangsu (*n* = 71,12.6%), Sichuan (*n* = 64, 11.4%), Shaanxi (*n* = 53, 9.4%), and Guangdong (*n* = 45, 8.0%) (Fig. S2) though this is of course open to sampling bias depending on the focus of the studies in which those genomes were created. The origin of isolates was specified for 556 of the 562 CPEn isolates with human (*n* = 496, 89.2%) accounting for the majority followed by animal (*n* = 33, 5.9%) and environment (*n* = 27, 4.9%), again likely due to substantial sampling bias.

The 562 CPEn isolates except 2 could be assigned to 113 STs including six new types, assigned ST3354, ST3355, and ST3357 to ST3360, after consulting with PubMLST (Data Set S1). The remaining two isolates could not be assigned to a ST as the *pyrG* gene was not detected in their genomes (GCA_023755165.1 and GCA_029310675.1). ST171 (*n* = 45) was the most common ST followed by ST418 (*n* = 43), ST78 (*n* = 33), ST88 (*n* = 33), ST93 (*n* = 25), and ST127 (*n* = 23) (Fig. S2). Of the 562 CPEn isolates, 517 (92%) encode a single carbapenemase with the remaining 45 (8.0%) genomes encoded two. NDM was the most common carbapenemase seen in 477 (84.9%) CPEn isolates, followed by IMP (in 101 isolates, 18.0%), KPC (in 16, 2.8%), VIM (in 9, 1.6%), OXA-48 (in three, 0.5%), and a rare Ambler class B carbapenemase, SIM (in one) (Fig. S2). As for specific variants, NDM-1 (*n* = 368, 65.5%) and NDM-5 (*n* = 100, 17.8%) were the most common, followed by IMP-4 (*n* = 61, 10.9%), IMP-26 (*n* = 24, 4.3%), KPC-2 (*n* = 14, 2.5%), and IMP-1 (*n* = 11, 2.0%). Notably, the vast majority (*n* = 549, 97.7%) encode at least one metallo-β-lactamase (MBL).

### The intra-hospital transmission and trans-national movement of ST171 CPEn

**Fig 2:**
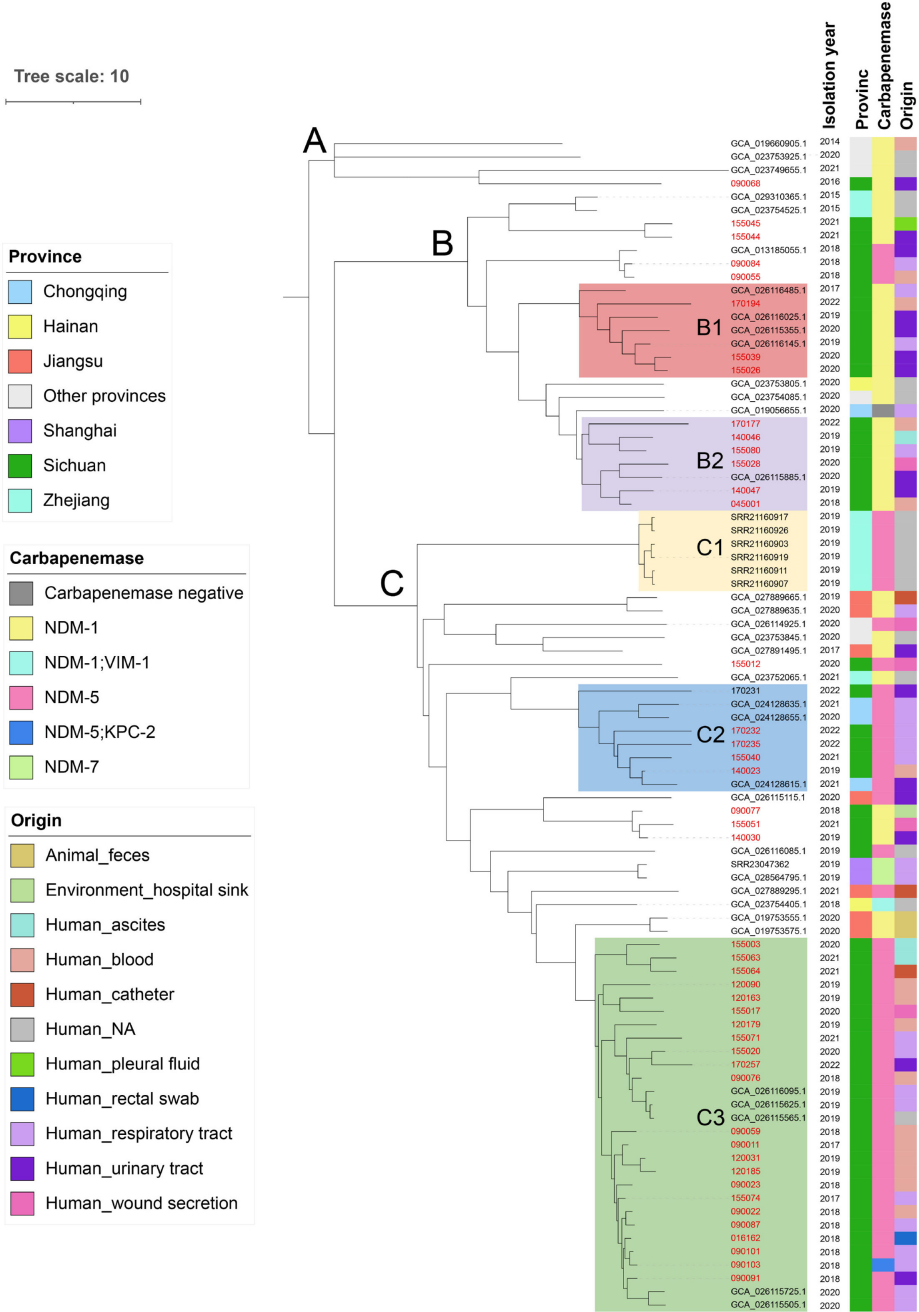
The phylogenomic tree of ST171 *Enterobacter* in China. A pseudo alignment of 88 ST171 Enterobacter genome was generated using the snippy-core script and from which a phylogenomic tree was inferred using IQ-TREE v2.2.2.6 with GTR + GAMMA model and 1,000 bootstraps, ignoring recombination sites identified by Gubbins v3.3.2. The tree is visualized using iTOL v6.9.1. Isolates from West China Hospital are depicted in red. Colors of strips represent different provincial regions of isolation, carbapenemases, and origins. Notably, three other ST171 isolates encoding a carbapenemase other than NDM were not included due to the divergent clonal background with over 20,000 SNPs.

We then focused on ST171, the most common type of CPEn in our collection and also in China as a whole, with a total of 91 from GenBank (*n* = 46) and our study (*n* = 45). These ST171 CPEn isolates were recovered between 2013 and 2022 and were seen in 12 provinces across China. With the exception of three isolates encoding either IMP-26 or VIM-1 (accession no. GCA_019449085, GCA_026114725, and GCA_030939715) or without any known carbapenemases (accession no. GCA_019056655), all other 87 ST171 CPEn encoded NDM, with NDM-5 (*n* = 50) being the predominant variant followed by NDM-1 (*n* = 35) and NDM-7 (*n* = 2). There were two isolates encoding two separate carbapenemases in combination, either NDM-1 plus VIM-1 (accession no. GCA_023754405) or NDM-5 plus KPC-2 (accession no. JAYGOH000000000). We found that the three isolates encoding non-NDM carbapenemases were separated by over 17,000 SNPs from the other 88 genomes, representing a distinct clonal background of the non-NDM strains. In contrast, the 88 ST171 isolates were separated by a maximum of 257 pairwise SNPs (Data Set S2). We inferred a phylogenomic tree of the 88 Chinese NDM-encoding ST171 isolates, annotated with their geographic location of isolation, carbapenemases, and origins (Fig. 2).

From the phylogenomic tree, we identified three well-supported branches (A, B, and C), which appeared to emerge successively with branching of A, B, and C, being dated to 2014, 2015, and 2017, respectively. Branch A comprises four isolates, all encoding NDM-1 and seen in different provincial regions. Branch B consists of 24 isolates mostly encoding NDM-1 (*n* = 20; other three encoding NDM-5 and one encoding no known carbapenemases) and can be further divided into several clades. Notably, each of two clades (B1 and B2) encompassed seven isolates from Sichuan and were separated by 0–25 and 0–33 intra-clade pairwise SNPs, respectively (Data set S2). Branch C is the largest clade comprising 60 isolates, most of which (*n* = 47) encode NDM-5 with the remaining encoding NDM-1 (*n* = 11) or NDM-7 (*n* = 2). Isolates of clade C1 (*n* = 6) were all positive for NDM-5 and recovered from Zhejiang (specifically, Wenzhou City) on 30 September 2019, with 0–2 SNPs, indicating sequencing of a clonal outbreak there. Clade C2 (*n* = 8) contained isolates all positive for NDM-5 and recovered from Sichuan or Chongqing, a provincial region neighboring Sichuan, with 0–51 SNPs. Isolates from our hospital were mainly located in Branch C, especially clade C3. Strains of clade C3 (*n* = 28) were all from Sichuan and positive for NDM-5 with 0–31 SNPs, and among them, 23 isolates were collected in our hospital as well as five strains isolated at another hospital in the same city (Chengdu, Sichuan).

To investigate the possible transmission of ST171 isolates, we calculated the substitution rate, which is 3.31 (95% highest posterior density [HPD], 2.97 to 3.68) SNPs per year with substitutions of 7.1 × 10^−7^ per nucleotide site per year. This estimation is consistent with an epidemiological study of 106 ST171 *E. xiangfangensis* (originally stated as *E. cloacae* complex) in the USA, which estimated 2.7 SNPs per year and 6.0 × 10^−7^ per nucleotide site per year (2). As such, we set a cutoff of 8 SNPs (twofold of the upper 95% HPD of the annual substitution rate as abovementioned) multiplied by the number of years between any two isolates to reconstruct most parsimonious transmission clusters.

**Fig 3:**
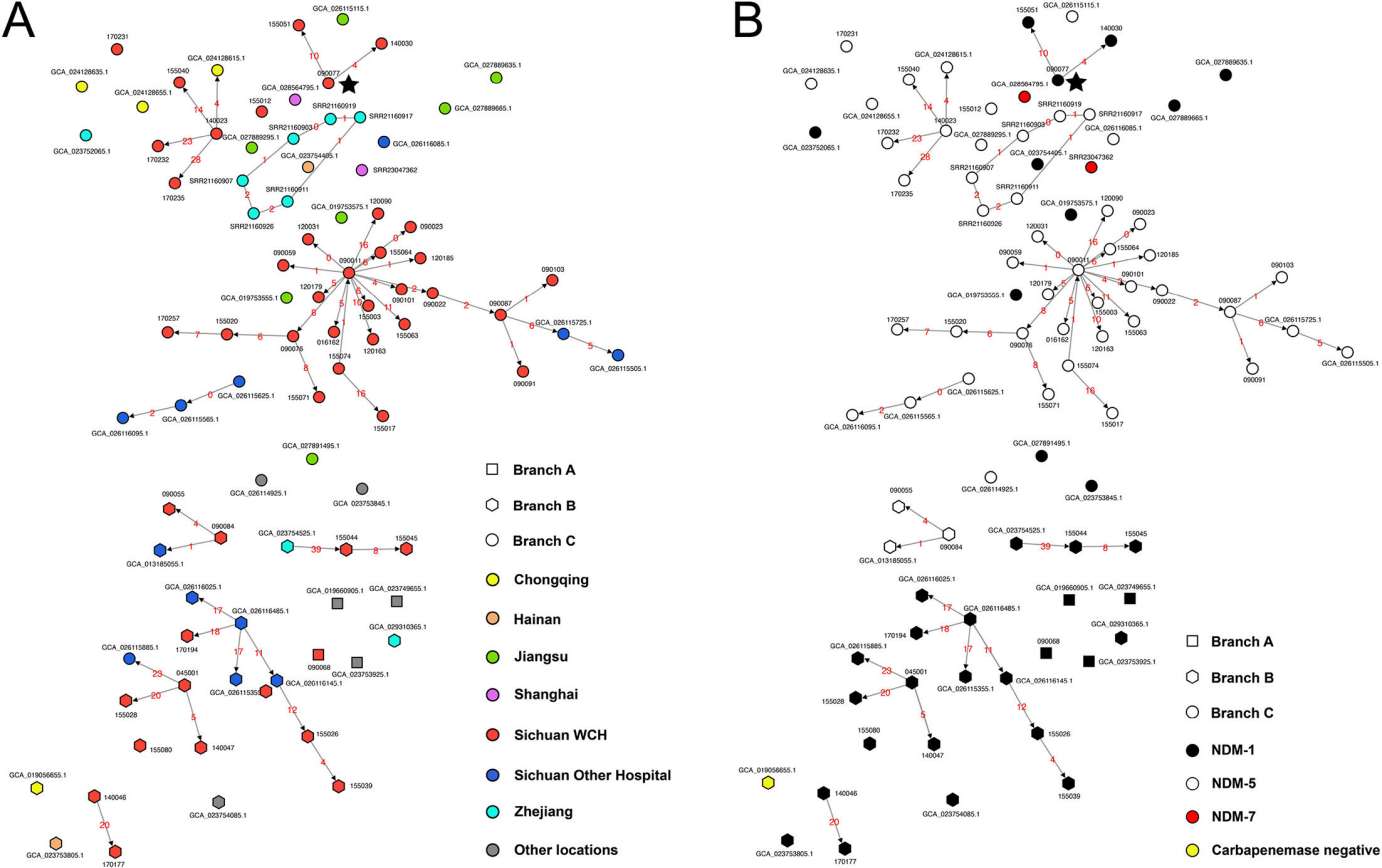
Transmission clusters of ST171 CPEn in China. This most parsimonious transmission diagram is based on SeqTrack algorithm using GraphSNP. Isolates within 8 × N SNPs, where 8 represents the maximum number of observed recombination-free SNPs per year multiplied by the number (N) of years between the isolates, are connected, with number of SNPs labeled in red. Orientation of arrows represents order of isolation time. Isolate names or accession numbers are displayed around the nodes. (A) Color of nodes represents the provincial region of isolation. (B) Color of nodes represents the encoded carbapenemase. Isolates of branch A, B, and C are shown by squares, hexagons, and circles, respectively. The isolate from a hospital sink is marked with a star.

**Fig 4:**
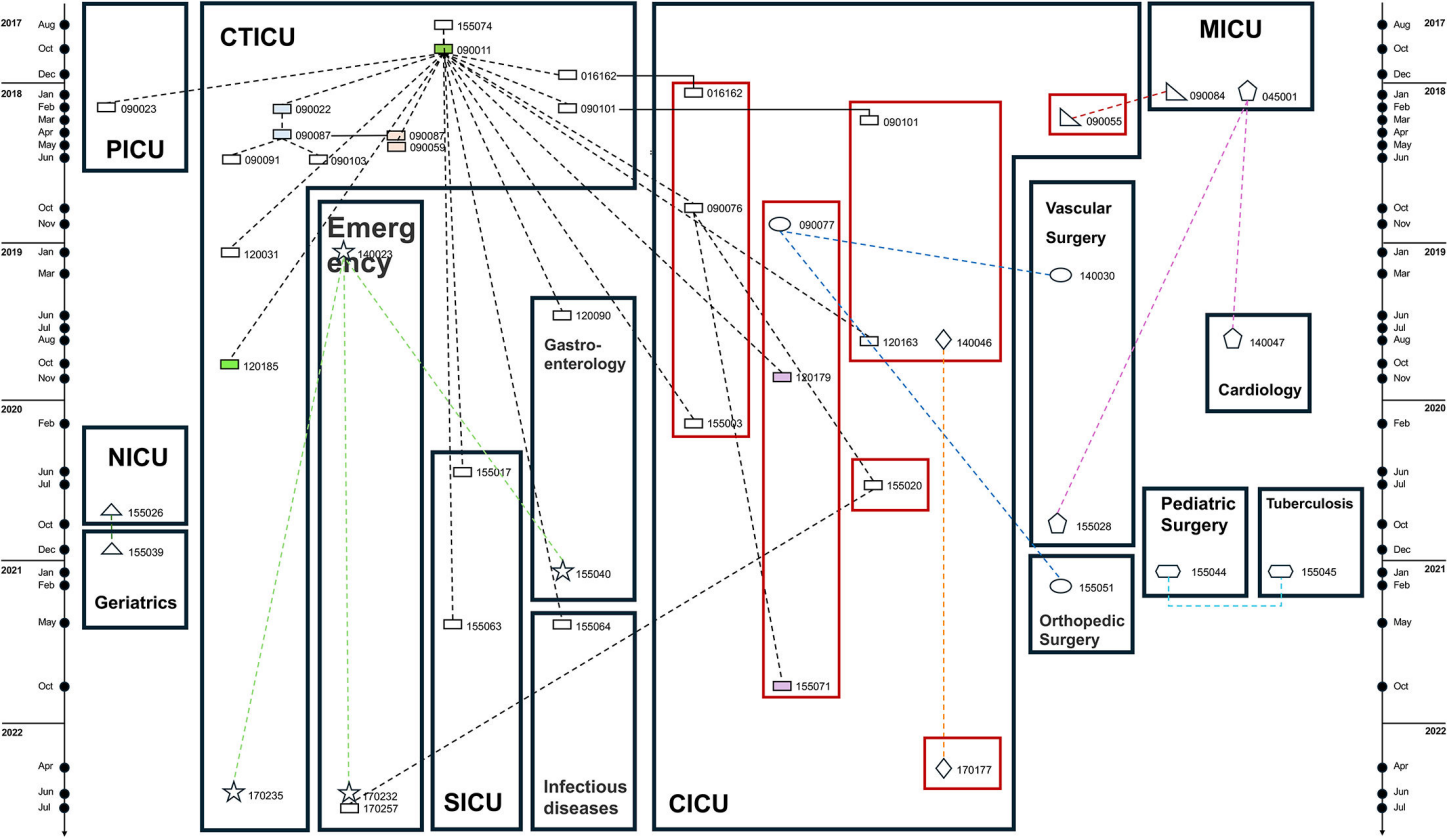
Spatiotemporal connections of ST171 CPEn isolates belonging to transmission clusters in the hospital. Timeline in the left is shown with years and month abbreviations. Transmission clusters are depicted in different forms such as circles, rectangles, stars, and triangles with isolate name being shown. Solid line represents the same patient to show the patient transfer. Dotted lines represent the transmission of different isolates of the same cluster. Abbreviation: CICU, center intensive care unit; CTICU, cardiothoracic intensive care unit; NICU, neurosurgical intensive care unit; MICU, medical intensive care unit; PICU, pediatric intensive care unit; SICU, surgical intensive care unit.

In our hospital, we identified 23 out of 45 ST171 CPEn isolates, all harboring *bla*_NDM-5_, belonging to a common transmission cluster (Fig. 3). These isolates were recovered between August 2017 and July 2022 from patients who were hospitalized in the Cardiothoracic ICU (CTICU, *n* = 9), Central ICU (*n* = 8), Surgery ICU (*n* = 2), or other four wards (Emergency, Gastroenterology, Infectious Diseases, and Pediatric ICU) (Fig. 4). Isolate 155074 is the first one (recovered in August 2017) in the transmission cluster, but most transmissions were linked to isolate 090011 (Fig. 3), which was recovered 2 months later from the same ward with a single SNP. Strikingly, 090011 is linked to 14 transmission events involving 7 wards (Fig. 3), among which are two transmission chains comprising multiple isolates with one chain containing four isolates in a single ward (CTICU) and another including four from two other wards (CICU and Emergency) (Fig. 4). Notably, the first isolate belonging to the transmission cluster in CICU was detected from a patient who was transferred from CTICU in January 2018 (Fig. 4), suggesting a possible patient-transfer-driven dissemination. Unfortunately, this cannot be definitively confirmed due to the absence of simultaneous environment sampling. In addition to the large transmission cluster, we identified seven intra-hospital clusters, which comprised two to four isolates belonging to Branch B or C. Of note, isolate 090077 was isolated from a sink in the hospital and predated the two linked clinical isolates (140030 and 155051) (Fig. 3 and 4), suggesting that the sink is a reservoir of CPEn.

We then looked at transmission beyond our isolates to wider China. Based on SeqTrack, an algorithm for reconstruction of most plausible genealogy of isolates, we detected four inter-hospital transmission clusters originating from isolate 090087 (clade C3), 090084 (clade B), 045001 (clade B2), and an unnamed one (GCA_026116485.1, clade B1) in Sichuan. Three of the four clusters appeared to originate from our hospital, while the remaining one is likely to have been introduced from another local hospital. We also unveiled two cross-regional transmissions originating from strain 140023 (from Sichuan to Chongqing) of clade C2 and an unnamed strain (GCA_023754525.1, from Zhejiang to Sichuan) of clade B (Fig. 3).

## DISCUSSION

In this study, we identified and genome sequenced 128 CPEn isolated from patients in West China Hospital between 2016 and 2022. Interrogation of the 14,648 publicly available *Enterobacter* genomes discovered a further 562 CPEn isolates from mainland China. Similar to isolates from our hospital, most publicly available CPEn from mainland China belonged to *E. xiangfangensis*, followed by *E. hoffmannii*. A multicenter epidemiological study in China (23) also found that *E. xiangfangensis* was the most common clinical carbapenem-resistant *Enterobacter* species (71.93%). *E. xiangfangensis* is often included in *E. hormaechei* and some studies (24–27) have identified that ST93, ST133, ST171, ST177, and ST418, all of which were claimed as *E. hormaechei* but actually belong to *E. xiangfangensis*.

In China, CPEn is very diverse in its clonal background as evidenced by the presence of many sequence types. Nevertheless, ST171 *E. xiangfangensis* was the most common CPEn type in China (23, 28) and is also a globally distributed lineage (29, 30). It appears that ST171 CPEn emerged in China more than once according to our phylogenomic analysis. The first ST171 CPEn isolate in China with a genome deposited in GenBank was recovered in 2013 encoding IMP-26 but did not widely disseminate across the country. In contrast, the first NDM-encoding ST171 CPEn isolate in China appeared in 2014 and has been found across China and further diverged into multiple clades and subclades. In particular, there were multiple ST171 clusters, all encoding NDM, circulating in our hospital and causing intra- and inter-ward transmission and also inter-hospital and cross-region transmission in China. As a high-risk lineage ST171 warrants further investigation to understand the genomic and microbiological factors contributing to its success.

The other common STs found in our local insolates (ST51, ST116, ST114, and ST97) differed from the other dominant STs across mainland China (ST418, ST78, ST88, ST93, and ST127). ST97 and ST78 belong to *E. hoffmannii,* and all others are *E. xiangfangensis*. Notably, many publicly available *Enterobacter* genomes originate from hospital outbreaks (31–33), and it has been previously found that the prevalent *Enterobacter* STs vary across regions of China (23). For instance, Zhou et al. (33) have reported that hypervirulent and carbapenem-resistant ST133 *E. hormaechei* (actually *E. xiangfangensis*) emerged in a tertiary hospital in Shenzhen (Southern China), while in Shenyang (Northeast China), carbapenem-resistant ST93 *E. xiangfangensis* was predominant (27).

We detected that the vast majority of CPEn from mainland China encode a MBL with a predominance of NDM. This differs from the USA where the serine-carbapenemase KPC is dominant (2, 34). NDM-1 accounts for the majority of MBLs both in our hospital and in mainland China (23, 28). Self-transmissible *bla*_NDM_-harboring IncX3-type plasmid was the most detected plasmid type, probably owing to its lower fitness cost than other types such as IncFII-type (23).

We found that approximately 2/3 of the CPEn isolates collected from our hospital were clinical isolates causing a variety of infections with bloodstream infection, pneumonia, and urinary tract infection the most common, and the majority of which were healthcare associated. The remaining 1/3 isolates were obtained from patients screening and colonized predominantly the respiratory tract without resulting in infections. Indeed, CPEn in respiratory samples was more commonly associated with colonization rather than infection. Several studies have reported an all-cause in-hospital mortality rate of infections due to carbapenem-resistant *Enterobacter*, which may contain isolates without carbapenemases, to be 9.4% (35), 14.3% (36), or 46% (37). The mortality rate of *Enterobacter* infections is impacted by multiple factors such as disease severity, host immune status, infection sites, clinical management, and bacterial species. Our predicted all-cause in-hospital mortality rate due to CPEn infections was 32.9%. This high mortality rate in our study may be due to a high proportion of bloodstream infection (37.6%) and the inclusion of patients discharged of their own will but predicted to die. Nevertheless, the high mortality highlights that CPEn presents a severe threat to infected patients.

As ST171 (of *E. xiangfangensis*) is the most common CPEn type in mainland China, we performed a focussed phylogenomic analysis on this lineage. We established a cutoff (8 SNPs) based on the calculated annual nucleotide substitution rate to define transmission clusters, which could be used to identify multiple intra-hospital hospital and several inter-hospital and cross-region transmissions. By analyzing the transmission of CPEn isolates in our hospital, we identified that intra-ward transmission was related to sharing patient rooms, while inter-ward transmission was mediated by patient transfer. Such findings are consistent with our previous observation for carbapenem-resistant *Klebsiella pneumoniae* in a study of a newly-open ICU (38). This highlights that environmental hygiene including sink cleaning may be key to address intra-ward transmission (39) and the transfer of patients with carbapenem-resistant *Enterobacterales* should be carefully managed and risk-assessed. For patients who have to be transferred, stringent control measures such as increased frequency of environment hygiene, contact precaution with rigorous monitoring of compliance, and the priority to be placed in single-room isolation (40) should be implemented.

Our study has some limitations. First, it is based on CPEn isolates from a single hospital in 6 years, which has intrinsic biases and limitations for generalization and may not fully reflect the recent changes in CPEn epidemiology. However, West China Hospital is a national medical hub receiving patients from across the country, resulting in increased representativeness of patients. Second, we analyzed CPEn genomes from NCBI. Publicly available genomes can result in sampling bias and may not reflect the real prevalence and population structure of CPEn. We purposefully did not aim to perform an epidemiological study but rather examined all available *Enterobacter* genomes (>14,000), from all reported sources, allowing the identification of high-risk clones and their dissemination. Third, we did not systematically collect samples from the hospital environment, which may underestimate the role of hospital environment as a reservoir facilitating onward dissemination. Nevertheless, we used the SeqTrack algorithm to attempt to reconstruct the most likely genealogy of isolates, allowing for an assessment of the spatiotemporal dynamics of their spread even in lack of environment sampling.

In conclusion, CPEn-related infection is typically healthcare associated and leads to high mortality, representing a severe clinical problem requiring effective infection control practice. There is a complicated CPEn population structure comprising multiple species and a large diversity of STs. ST171 *E. xiangfangensis* encoding NDM is the major high-risk CPEn lineage in China. In addition, multiple in-hospital transmissions possibly associated with sharing the same room or bed and inter-ward patient transfer and several inter-hospital transmissions were observed for ST171 CPEn, which requires rigorous surveillance and further studies as to its true prevalence and clinical impact.

## SUPPLEMENTAL MATERIAL

Supplemental Material spectrum.01166-25-s0001.pdf Figures S1 and S2; Table S1.

Data Set S1 spectrum.01166-25-s0002.xlsx Characteristics of the 562 CPEn strains from China in GenBank.

Data Set S2 spectrum.01166-25-s0003.xlsx Pairwise SNPs between ST171 strains.

ASM does not own the copyrights to Supplemental Material that may be linked to, or accessed through, an article. The authors have granted ASM a non-exclusive, world-wide license to publish the Supplemental Material files. Please contact the corresponding author directly for reuse.
