# Supplementary material for: The clinical impact and dissemination of carbapenemase-producing Enterobacter: a genome-based study in China
Source: Microbiol Spectr. 2025 Aug 12;13(9):e01166-25. doi: 10.1128/spectrum.01166-25 (PMC12403762; doi:10.1128/spectrum.01166-25)
Supplement: Supplemental Material — Figures S1 and S2; Table S1. [file spectrum.01166-25-s0001.pdf]

Supplementary files

Supplemental Figure S1. Flowchart of inclusion criteria for *Enterobacter* assemblies and SRA from GenBank.

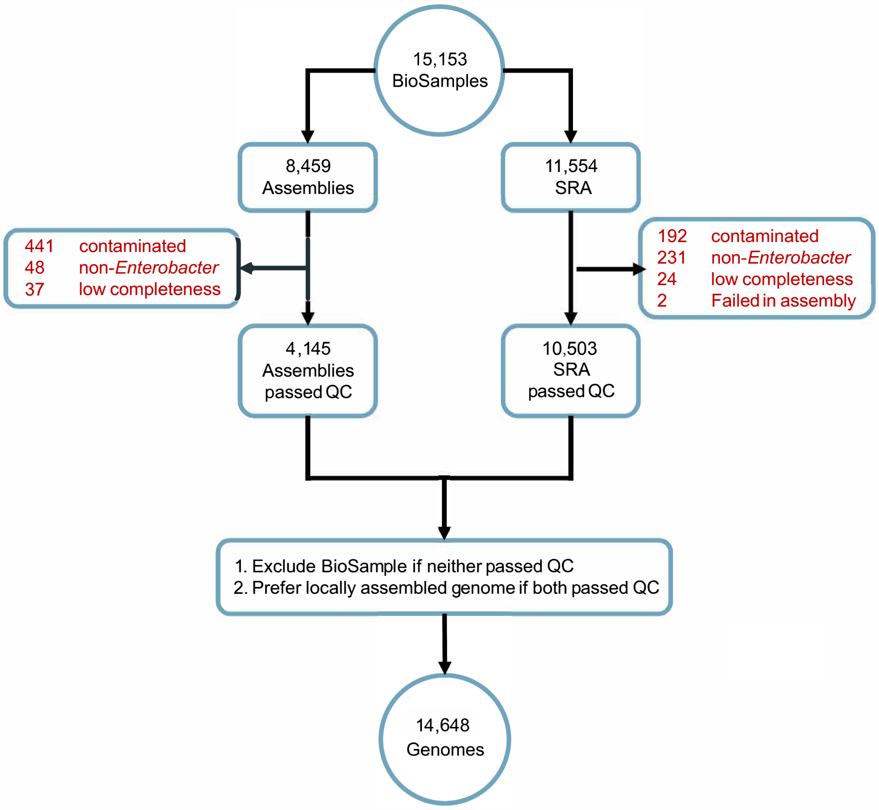

Supplementary Figure S2. Characteristics of 562 CPEn in China. (A) Precise species identification using FastANI. (B) Sources of the CPEn. (C) Sequence type distribution of the CPEn. (D) Carbapenemases identified by AMRFinder.

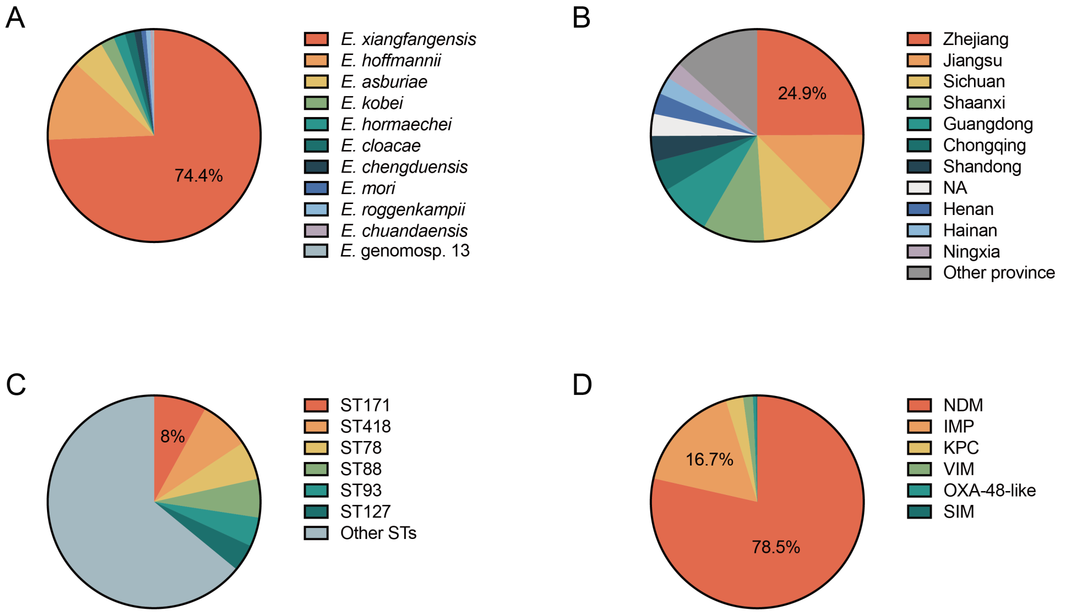

Table S1 Type strains of *Enterobacter* Genus

Species	Type strains	Accession number	Reference
Enterobacter asburiae	ATCC 35953	CP011863.1	[1]
Enterobacter bugandensis	EB-247	LT992502.1	[2]
Enterobacter cancerogenus	ATCC33241	FYBA01000005.1	[3]
Enterobacter chengduensis	WCHECl-C4	CP043318.1	[4]
Enterobacter chuandaensis	90028	QZCS01000013.1	[5]
Enterobacter cloacae	DSM 30054	CP056776.1	[6]
Enterobacter dissolvens	ATCC 23373	WJWQ01000007.1	[1]
Enterobacter dykesii	E1	VTTY01000013.1	[7]
Enterobacter hoffmannii	DSM 14563	CP017186.1	[8]
Enterobacter hormaechei	ATCC 49162	GL892086.1	[9]
Enterobacter huaxiensis	90008	QZCT01000004.1	[5]
Enterobacter kobei	DSM 13645	CP017181.1	[10]
Enterobacter ludwigii	EN-119	CP017279.1	[11]
Enterobacter mori	LMG 25706	GL890773.1	[12]
Enterobacter oligotrophica	CCA6	AP019007.1	[13]
Enterobacter quasihormaechei	WCHEQ120003	SJON01000012.1	[14]
Enterobacter quasimori	90044	RXRX01000010.1	[15]
Enterobacter quasiroggenkampii	WCHECL1060	LFDQ02000008.1	[15]
Enterobacter roggenkampii	DSM 16690	CP017184.1	[8]
Enterobacter sichuanensis	WCHECL1597 14	POVL01000014.1	[16]
Enterobacter soli	ATCC BAA-2102	LXES01000018.1	[17]
Enterobacter vonholyi	E13	VTUC01000012.1	[7]
Enterobacter wuhouensis	WCHEW120002	SJOO01000006.1	[14]
Enterobacter xiangfangensis	LMG 27195	CP017183.1	[18]

References

[1] Brenner D J, Mcwhorter A C, Kai A, et al.*Enterobacter asburiae* sp. nov., a new species found in clinical specimens, and reassignment of Erwinia dissolvens and Erwinia nimipressuralis to the genus *Enterobacter* as *Enterobacter dissolvens* comb. nov. and *Enterobacter nimipressuralis* comb. nov[J].J Clin Microbiol,1986, 23 (6): 1114-20.

[2] Doijad S, Imirzalioglu C, Yao Y, et al.*Enterobacter bugandensis* sp. nov., isolated from neonatal blood[J].Int J Syst Evol Microbiol,2016, 66 (2): 968-974.

[3] Schønheyder H C, Jensen K T, Frederiksen W.Taxonomic notes: synonymy of *Enterobacter cancerogenus* (Urosević 1966) Dickey and Zumoff 1988 and *Enterobacter taylorae* Farmer et al. 1985 and resolution of an ambiguity in the biochemical profile[J].Int J Syst Bacteriol,1994, 44 (3): 586-7.

[4] Wu W, Feng Y, Zong Z.Characterization of a strain representing a new Enterobacter species, *Enterobacter chengduensis* sp. nov[J].Antonie Van Leeuwenhoek,2019, 112 (4): 491-500.

[5] Wu W, Wei L, Feng Y, et al.*Enterobacter huaxiensis* sp. nov. and *Enterobacter chuandaensis* sp. nov., recovered from human blood[J].Int J Syst Evol Microbiol,2019, 69 (3): 708-714.

[6] Hormaeche E, Edwards P.A proposed genus *Enterobacter*[J].International Bulletin of Bacteriological Nomenclature and Taxonomy,1960, 10 (2): 71-4.

[7] Cho G-S, Stein M, Fiedler G, et al.Polyphasic study of antibiotic-resistant enterobacteria isolated from fresh produce in Germany and description of *Enterobacter vonholyi* sp. nov. isolated from marjoram and *Enterobacter dykesii* sp. nov. isolated from mung bean sprout[J].Syst Appl Microbiol,2021, 44 (1): 126174.

[8] Sutton G G, Brinkac L M, Clarke T H, et al.*Enterobacter hormaechei* subsp. *hoffmannii* subsp. nov., *Enterobacter hormaechei* subsp. *xiangfangensis* comb. nov., *Enterobacter roggenkampii* sp. nov., and *Enterobacter muelleri* is a later heterotypic synonym of *Enterobacter asburiae* based on computational analysis of sequenced *Enterobacter* genomes[J].F1000Res,2018, 7: 521.

[9] O'hara C M, Steigerwalt A G, Hill B C, et al.*Enterobacter hormaechei*, a new species of the family *Enterobacteriaceae* formerly known as enteric group 75[J].J Clin Microbiol,1989, 27 (9): 2046-9.

[10] Kosako Y, Tamura K, Sakazaki R, et al.*Enterobacter kobei* sp. nov., a new species of the family *Enterobacteriaceae* resembling *Enterobacter cloacae*[J].Curr Microbiol,1996, 33 (4): 261-5.

[11] Hoffmann H, Stindl S, Stumpf A, et al.Description of *Enterobacter ludwigii* sp. nov., a novel *Enterobacter* species of clinical relevance[J].Syst Appl Microbiol,2005, 28 (3): 206-12.

[12] Zhu B, Lou M M, Xie G L, et al.*Enterobacter mori* sp. nov., associated with bacterial wilt on Morus alba L[J].Int J Syst Evol Microbiol,2011, 61 (Pt 11): 2769-2774.

[13] Akita H, Matsushika A, Kimura Z I.*Enterobacter oligotrophica* sp. nov., a novel oligotroph isolated from leaf soil[J].Microbiologyopen,2019, 8 (9): e00843.

[14] Wang C, Wu W, Wei L, et al.*Enterobacter wuhouensis* sp. nov. and *Enterobacter quasihormaechei* sp. nov. recovered from human sputum[J].Int J Syst Evol Microbiol,2020, 70 (2): 874-881.

[15] Wu W, Feng Y, Zong Z.Precise species identification for *Enterobacter*: a genome sequence-based study with reporting of two novel species, *Enterobacter quasiroggenkampii* sp. nov. and *Enterobacter quasimori* sp. nov[J].MSystems,2020, 5 (4): e00527-20.

[16] Wu W, Feng Y, Zong Z.*Enterobacter sichuanensis* sp. nov., recovered from human urine[J].Int J Syst Evol Microbiol,2018, 68 (12): 3922-3927.
[17] Manter D K, Hunter W J, Vivanco J M.*Enterobacter soli* sp. nov.: a lignin-degrading γ-proteobacteria isolated from soil[J].Curr Microbiol,2011, 62 (3): 1044-9.
[18] Gu C T, Li C Y, Yang L J, et al.*Enterobacter xiangfangensis* sp. nov., isolated from Chinese traditional sourdough, and reclassification of Enterobacter sacchari Zhu et al. 2013 as *Kosakonia sacchari* comb. nov[J].Int J Syst Evol Microbiol,2014, 64 (Pt 8): 2650-2656.
